# CRISPR signal conductor 2.0 for redirecting cellular information flow

**DOI:** 10.1038/s41421-021-00371-1

**Published:** 2022-03-15

**Authors:** Yonghao Zhan, Aolin Li, Congcong Cao, Yuchen Liu

**Affiliations:** 1 Guangdong Key Laboratory of Systems Biology and Synthetic Biology for Urogenital Tumors, Shenzhen Institute of Translational Medicine, Health Science Center, The First Affiliated Hospital of Shenzhen University, Shenzhen Second People’s Hospital, Shenzhen, Guangdong China; 2 Department of Urology, The First Affiliated Hospital of Zhengzhou University, Zhengzhou, Henan China; 3 Department of Urology, Shenzhen Second People’s Hospital, The First Affiliated Hospital of Shenzhen University, International Cancer Center, Shenzhen University School of Medicine, Shenzhen, Guangdong China

**Keywords:** Biological techniques, Cancer therapy, Long non-coding RNAs

## Abstract

A key challenge in designing intelligent artificial gene circuits is generating flexible connections between arbitrary components and directly coupling them with endogenous signaling pathways. The CRISPR signal conductor based on conditionally inducible artificial transcriptional regulators can link classic cellular protein signals with targeted gene expression, but there are still problems with multiple signal processing and gene delivery. With the discovery and characterization of new Cas systems and long noncoding RNA (lncRNA) functional motifs, and because of the compatibility of guide RNA with noncoding RNA elements at multiple sites, it is increasingly possible to solve these problems. In this study, we developed CRISPR signal conductor version 2.0 by integrating various lncRNA functional motifs into different parts of the crRNA in the CRISPR-dCasΦ system. This system can directly regulate the expression of target genes by recruiting cellular endogenous transcription factors and efficiently sense a variety of protein signals that are not detected by a classical synthetic system. The new system solved the problems of background leakage and insensitive signaling responses and enabled the construction of logic gates with as many as six input signals, which can be used to specifically target cancer cells. By rewiring endogenous signaling networks, we further demonstrated the effectiveness and biosafety of this system for in vivo cancer gene therapy.

## Introduction

In living organisms, cells respond to diverse biological signals through natural gene circuits^1^, and timely feedback improves their ability to adapt to complex external environments. From relatively simple bistable switches^2^, biological oscillators^3,4^ and Boolean logic gates^5^ to more complex hierarchical gene regulation networks^6^, an important direction in synthetic biology is construction of artificial gene circuits that can autonomously perform user-defined biological functions by integrating tiny biological components^7^. Artificial gene circuits can be used to rewire the metabolic system of microorganisms, thus enabling host cells to synthesize specific chemicals^8^ and directing mammalian cells to sense specific disease signals and synthesize artificial drugs^9^. Almost all human diseases, especially cancers, are triggered by dysregulation of natural gene regulatory networks caused by gene mutations, abnormal gene expression, or alternative splicing of transcripts^10^. Synthetic gene circuits sense the overall expression pattern of a group of cancer-promoting and tumor-suppressing factors, including microRNAs (miRNA), mRNAs, transcription factors, and RNA-binding proteins, to determine the benign and malignant states of cells and specifically activate apoptotic genes to kill cancer cells without affecting normal cells^11,12^. Synthetic gene circuits can be transfected through plasmid vectors into cell lines cultured in vitro. They can also be delivered via viral vectors and lipid nanomaterials for in vivo gene therapy and therefore have potentially broad applications^13^.

Although intelligent artificial gene circuits have been established, composability remains a challenge, specifically the ability to connect arbitrary components and directly couple them with endogenous signaling pathways. To solve this problem, two main strategies have been developed; in one solution, protein–protein interactions are engineered^14,15^, and in the other solution, RNA-based riboregulators are designed^16,17^. Combining protein–protein regulatory systems can produce a variety of circuit structures, which facilitate rational gene circuit design, and through their interactions, proteins can respond to signals very quickly^18^. However, heterogeneous proteins are more likely to cause immune responses in the body, which are not conducive to clinical applications. RNA devices can sense RNA molecules such as miRNAs and mRNAs through antisense strategies based on complementary pairing and can also bind cellular proteins through artificially screened or natural aptamers. However, RNAs can be unstable and easily degraded, and it may be necessary to build a library to screen RNAs with the best regulatory effects on target genes^19^.

The bacterial type II clustered regularly interspaced short palindromic repeat (CRISPR)*–*CRISPR-associated nuclease 9 (Cas9) system provides a modular tool for genome editing and regulation^20^. The wild-type or modified Cas protein binds to target DNA through a guide RNA (gRNA) and then cuts or epigenetically modifies the target sequence^21^. Since the programmability of the CRISPR system was first reported, it has been widely used in various genetic engineering studies. For example, the CRISPR-Cas protein was used as an artificial transcription factor to achieve transcriptional control of genes of interest^22,23^. Compared with the use of the CRISPR system to inhibit gene transcription, the CRISPR gene activation system can upregulate the expression of endogenous genes. To further improve RNA regulation of endogenous genes and related signaling pathways, our group constructed a CRISPR signal conductor^24^ by introducing an RNA aptamer-modified riboswitch into a single guide RNA (sgRNA) in conjunction with CRISPR-endonuclease dead Cas9 (dCas9). The regulatory network between different cellular protein signals can be reprogrammed through this approach, and we further constructed an internal tumor suicide system by linking pro-tumor signaling to the activation of anti-tumor signaling pathways and then used the system to kill various tumor cells, including bladder cancer cells. Since then, many other groups have published similar reports showing how they further modified sgRNA and developed inducible CRISPR systems^25–28^, for example, by introducing ligand-activated self-cleaving ribozymes into sgRNA to regulate its activity^25^. Some researchers have modified the active domain of the Cas protein to control its gene regulation with small-molecule drugs or light exposure^29,30^. However, many problems with the original version of the CRISPR signal conductor still need to be solved, such as difficulty in integrating more than two input signals, relatively low transcriptional regulation activity, CRISPR activity leakage, few sensed signals due to the limited number of aptamers, and a gene delivery problem caused by the size of the CRISPR-dCas9 system. To resolve these problems, we and other research groups have developed novel transcription control systems based on different types of Cas nucleases^31^ and translational control systems based on *trans*-acting noncoding RNAs (ncRNAs)^32^. Specifically, we developed CRISPReader technology^33^ to initiate a promoter-less gene expression modality to simplify the CRISPR system and facilitate its delivery with adeno-associated virus (AAV) vectors. However, the other aforementioned challenges have not been adequately addressed to date.

With the development of genomics, many long noncoding RNAs (lncRNAs) have been discovered, and the functional regulatory mechanisms of these lncRNAs have been determined^34^. LncRNAs bind to the gene promoter region through antisense complementary sequences, and the molecular scaffold formed by the lncRNA secondary structure can combine with transcription factors and other proteins and direct them to the promoter region of target genes^35,36^. This gene regulatory mechanism is very similar to that of the CRISPR-Cas9/sgRNA complex^37^, which uses RNA–protein interactions to regulate gene expression. Because the protein-binding domain of lncRNAs is similar to that of an aptamer, an important question is whether the functional lncRNA motifs can be integrated into sgRNAs. CRISPR-Display technology^38^ can translocate an entire lncRNA to a specific DNA region using the Cas9/sgRNA complex. However, many lncRNAs contain more than 500 nucleotides (nt) and many complex secondary structures. Therefore, the specific functional lncRNA motifs identified in recent years need to be refined and incorporated into sgRNAs, which may simplify the CRISPR signal conductor system and enable its interaction with a larger number of cellular protein molecules.

The CRISPR-Cas system is typically found only in prokaryote genomes, but the latest research shows that the genomes of giant bacteriophages also contain sequences encoding the CRISPR system^39^. CasΦ is one of the Cas protein family members encoded by giant phages, and it is only 70–80 kDa, only approximately one-half the size of Cas9 or Cas12a^40^. It can be used for effective gene editing in both human and plant cells. The deactivated CasΦ variant (dCasΦ) system, similar to dCas9, may also regulate the expression levels of target genes by fusing with transcriptional regulatory factors. The gRNA of the CasΦ system is very simple, including only one hairpin structure and an antisense sequence, which is very suitable for combination with riboswitches. Therefore, the compact CasΦ system provides new possibilities for optimizing the size of gene editors and CRISPR signal conductors.

To solve the problems of previous CRISPR signal conductors, we developed the CRISPR signal conductor version 2.0 by integrating various lncRNA functional motifs into different parts of the CRISPR RNA (crRNA) in dCasΦ. This system can be used to regulate the expression of target genes through endogenous transcription factors and efficiently sense a variety of protein signals. It solves the problem of background leakage, and can be used to construct a logic gate system with as many as six input signals, which can specifically target cancer cells. After combination with CRISPReader technology, we demonstrated the possible effectiveness and biosafety of this system for in vivo gene therapy in cancers.

## Results

### Design of CRISPR signal conductor 2.0

In a previous study, lncRNAs were functionally appended onto the sgRNA of Cas9 at multiple positions, including the 5′-end, 3′-end, and middle stem-loop region of the sgRNA^38^. However, the locations and sizes of the insertions within the crRNA of dCasΦ need to be further explored. Because complete lncRNA is usually very long, we decided to integrate shorter functional lncRNA motifs that had been identified in previous studies into the crRNA to construct a crRNA–ncRNA fusion whose expression can be driven by the U6 RNA polymerase III promoter.

**Fig. 1:**
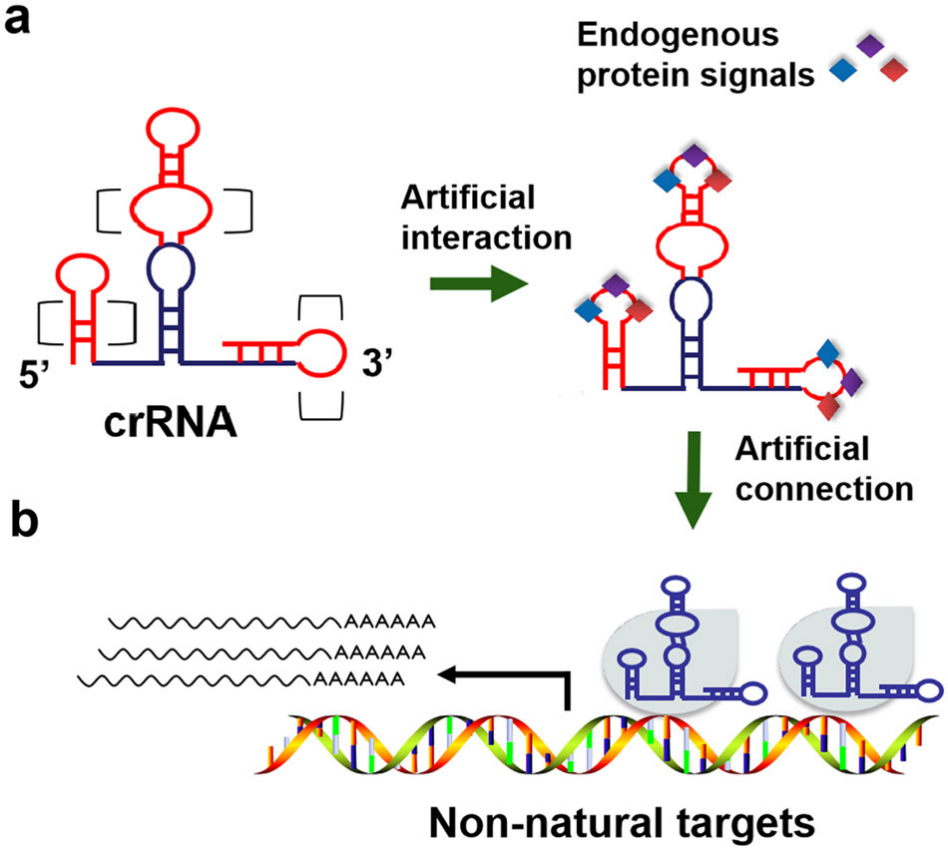
Design and construction of CRISPR signal conductor 2.0. **a** The possible insertion sites for lncRNA functional motifs in the crRNA of CRISPR-dCasΦ. The 5′-end, 3′-end, and intermediate stem-loop of crRNA were sequentially inserted with protein-binding RNA elements and a lncRNA functional motif-based riboswitch. **b** CRISPR signal conductor 2.0 bound different endogenous proteins, including transcriptional activators, transcriptional repressors, and RNA-binding proteins, through reprogrammed crRNA, and then regulated the expression of target genes in cells, forming a logical network controlled by multiple signals.

First, we analyzed the composition of gRNA of the CRISPR-CasΦ-2 system, which consisted of a 44-nt crRNA with a short hairpin structure for binding CasΦ protein and a target-specific antisense RNA fused to the 3′-end of the crRNA. Given the remarkable simplicity of the crRNA structure of CasΦ, we reasoned that lncRNA functional motifs could also be inserted into the crRNA scaffold in the three abovementioned locations (Fig. 1a). The lncRNA motifs that bind to various functional proteins, such as transcription factors, can be inserted into the 5′-end of crRNA to recruit endogenous transcriptional regulatory complexes, and riboswitch-containing protein-binding elements can be inserted into the DNA recognition region at the 3′-end to induce crRNA binding to target DNAs. In addition, the loop in the middle region of the crRNA can also be fused with some lncRNA motifs that bind to endogenous proteins to regulate crRNA activity. In contrast to the original CRISPR signal conductor, which depended only on a limited number of aptamers to sense proteins, CRISPR signal conductor 2.0 as described is based on numerous lncRNA domains that bind and regulate cellular endogenous signaling molecules (Fig. 1b).

### Regulation of gene expression based on endogenous transcription factors

**Fig. 2:**
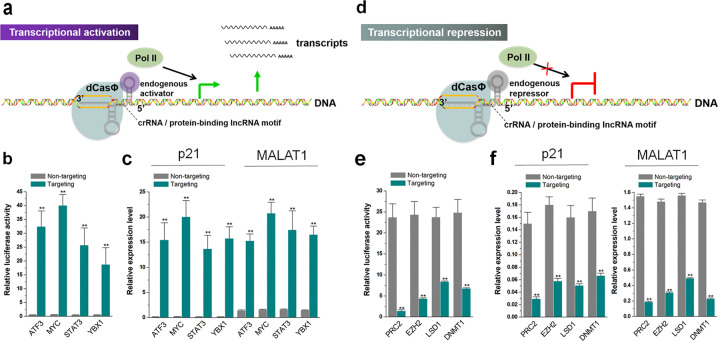
Endogenous transcription factors regulate gene activation/inactivation. **a** Schematic diagram showing the molecular mechanism underlying enhanced transcription of cellular genes accomplished by binding endogenous transcription activators. **b** The effect of gene activation was determined by dual-luciferase reporter assay. Relative luciferase activities were determined as the ratios between Rluc and Fluc values. **c** The relative expression levels of P21 and MALAT1 upregulated by endogenous transcription activators were determined by real-time quantitative PCR (RT-qPCR). **d** Schematic diagram showing the molecular mechanism leading to repressed transcription of cellular genes by binding endogenous transcription repressors. **e** The effect of gene inactivation was detected by the dual-luciferase reporter assay. **f** The relative expression levels of p21 and MALAT1 were downregulated by endogenous transcription repressors, compared to the control, as determined by RT-qPCR. The results are shown as the means ± SD. Each experiment was performed in triplicate for five independent times. *P < 0.05; **P < 0.01, compared to the control, two-tailed *t*-test.

To determine whether CRISPR-dCasΦ system can upregulate the expression of the genes of interest by fusing the crRNA with lncRNA functional motifs that can bind to transcription activators, we separately inserted the lncRNA motifs that bind to the transcription activators ATF3^41^, MYC^42^, STAT3^43^, and YBX1^44^ into the 5′-end of crRNAs to construct a series of transcription activation devices (Fig. 2a). We then evaluated the transcriptional activation ability of these devices in HEK-293 cells by using a dual-luciferase reporter system, which expresses both Renilla luciferase (Rluc) and firefly luciferase (Fluc) driven by different promoters in a single vector. Rluc, which is driven by a tetracycline responsive element (TRE) promoter, was chosen as the target of the designed crRNAs, with Fluc treated as the internal control. The crRNA/dCasΦ expression vector was transfected into HEK-293 cells stably expressing the dual-luciferase construct and the luciferase activity was measured 48 h post transfection. The results indicated that these four transcription activators all increased the relative expression level of Rluc to varying degrees (Fig. 2b), and the MYC-dependent transcription activator exhibited the best effect with an average increase of ~70-fold. To further verify the transcriptional activation effect, we selected two endogenous genes, the protein-coding gene P21 and lncRNA MALAT1, as target genes. We detected the expression of these genes in HEK-293 cells 48 h after transfection and found that the results were similar to those of the reporter genes (Fig. 2c). We also compared the transcriptional activators with the classical dCas9-VPR systems using matching gRNAs under the same transfection conditions and found that MYC-dependent transcriptional activators performed better than the dCas9-VPR designs, but other transcription factor-dependent transcriptional activators showed a weaker or comparable effect in comparison to dCas9-VPR (Supplementary Fig. S1). To further demonstrate that the abovementioned transcriptional activation effect was caused by the regulation of intracellular transcription factors, we manipulated the expression of transcription factors by overexpression and RNA interference (RNAi). We found that transfection of overexpression vectors enhanced the transcriptional effect, while RNAi produced the opposite effect (Supplementary Fig. S2). These results indicated that crRNA fused with lncRNA functional elements activated target gene expression by binding transcription activators and recruiting endogenous transcription systems.

To further determine whether the CRISPR-dCasΦ system can downregulate the expression of the genes of interest when crRNA was fused to lncRNA functional motifs that bind to transcription repressors, we separately inserted the lncRNA motifs that bind to the transcription repressors PRC2^45^, EZH2^46^, LSD1^47^, and DNMT1^48^ into the 5′-end of the crRNAs to construct a series of transcription repression devices (Fig. 2d). The transcriptional repression ability in HEK-293 cells was investigated using a dual-luciferase reporter system similar to that described above; in this case, Rluc was driven by the SV40 promoter. Forty-eight hours after transfection, the luciferase assay indicated that the four transcription repressor devices all suppressed the expression of the reporter gene to varying degrees (Fig. 2e). Among them, the device that relied on the PRC2 transcription repressor complex showed the best silencing effect, with ~94% decrease of luciferase activity. To further verify the transcriptional repression effect, we selected two endogenous genes as targets and found similar results to those of the reporter genes (Fig. 2f). We then compared these transcriptional repressors with classical dCas9-KRAB systems using matching gRNAs under the same transfection conditions and found that the effects of these transcription factor-dependent transcriptional repressors were comparable to those of dCas9-KRAB (Supplementary Fig. S3). The use of overexpression and RNAi confirmed the dependence of these devices on endogenous transcription repressors (Supplementary Fig. S4). Together, these results indicated that crRNA fused with lncRNA functional elements repressed target gene expression by binding endogenous transcription repressors.

### Inducible regulation of target gene expression by endogenous proteins

**Fig. 3:**
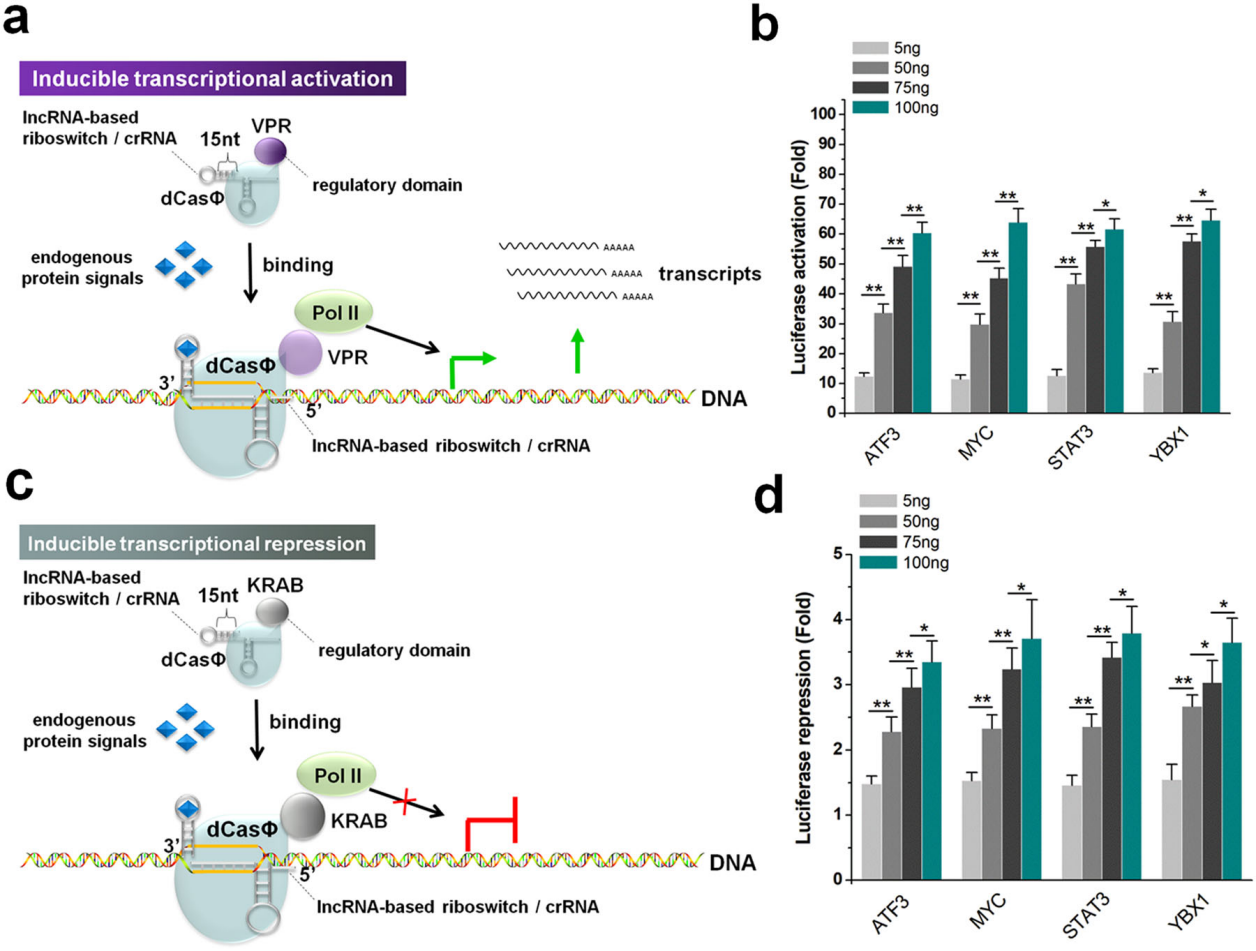
Protein signal-inducible transcriptional activation/inactivation. **a** Schematic diagram showing the molecular mechanism of transcription activation of cellular genes induced by endogenous proteins. The device linked an internal protein to the transcriptional activation of a downstream gene. **b** The effect of gene activation was detected by the dual-luciferase reporter assay. Relative luciferase activities were determined as the ratios of Rluc to Fluc values. The fold change in each group was determined by comparison with the crRNA negative control. **c** Schematic diagram showing the molecular mechanism of transcription repression of cellular genes induced by endogenous proteins. The device linked the presence of internal protein to the transcriptional inactivation of a downstream gene. **d** The effect of gene inactivation was detected by the dual-luciferase reporter assay. The fold change in each group was determined by comparison with the crRNA negative control. All values represent the means ± SD from five independent experiments. *P < 0.05; **P < 0.01, relative to the control, two-tailed *t*-test.

To further determine whether the CRISPR-dCasΦ system can influence gene expression in response to intracellular protein signals after fusion with a lncRNA functional motif-based riboswitch, we connected each of the lncRNA motifs that bind to cellular proteins ATF3, MYC, STAT3, and YBX1 with an antisense sequence complementary to the crRNA spacer and then respectively introduced them into the 3′-end of the crRNAs (Fig. 3a). The inducible transcriptional activation ability of the dCasΦ-VPR device was tested in HEK-293 cells expressing a dual-luciferase reporter system, in which Rluc was driven by the TRE promoter. To dynamically regulate the intensity of protein signals, different amounts of a corresponding protein overexpression plasmid were co-transfected into the cells. The luciferase assay results obtained 48 h after transfection showed that the relative expression levels of Rluc increased with increasing of the plasmid amounts (Fig. 3b). In contrast, the relative expression levels of Rluc gradually decreased as the concentration of short hairpin RNAs (shRNAs) targeting the corresponding protein-coding gene was increased 48 h after shRNA transfection (Supplementary Fig. S5). The inducible transcriptional repression ability of the dCasΦ-KRAB device (Fig. 3c) was also tested using a similar method in HEK-293 cells expressing the dual-luciferase reporter system, in which Rluc was driven by the SV40 promoter. The luciferase assay results obtained 48 h after transfection showed that the relative expression levels of Rluc decreased with increasing of amounts of transfected plasmid (Fig. 3d). The basic expression level of Rluc also gradually increased with increased shRNA concentrations (Supplementary Fig. S6). To further illustrate the universality of this strategy, we integrated other previously identified lncRNA sequences that bind to IGF2BP1, LIN28B, FTO, and TIA1^49^ into the crRNA riboswitch, and obtained similar results (Supplementary Fig. S7). Together, these results indicated that this approach effectively linked the expression of intracellular proteins to the transcriptional regulation of target genes.

### Dynamic and sensitive responses to endogenous signals

**Fig. 4:**
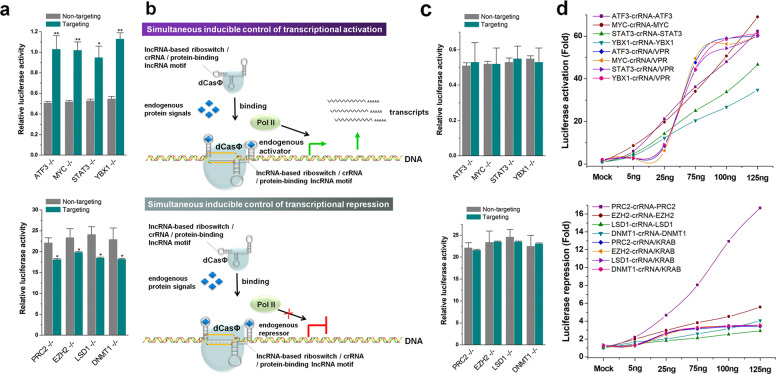
Dynamic and sensitive responses to endogenous protein signals. **a** The inducible dCasΦ-VPR and dCasΦ-KRAB systems showing basic transcriptional regulatory activity on Rluc. **b** Schematic diagram of the simultaneous inducible control of transcriptional activation. **c** The simultaneous inducible CRISPR transcriptional systems did not show any leakage. **d** The simultaneous inducible CRISPR systems showed a broader signal response range than the previous systems. The data were normalized to those of the non-targeting crRNA group and displayed as average percentages (%). Relative luciferase activities were determined as the ratios of Rluc to Fluc values. Error bars represent the SD from five independent experiments. *P < 0.05; **P < 0.01, relative to the control, two-tailed *t*-test.

Because the previously developed inducible CRISPR transcription regulation systems often showed a certain degree of leakage, we used the abovementioned HEK-293 cell lines stably transfected with luciferase to construct cell lines with knockout of specific endogenous proteins and then tested the inducible dCasΦ system. Forty-eight hours after transfection, the aforementioned inducible dCasΦ-VPR and dCasΦ-KRAB systems still showed basic transcriptional regulatory activity towards Rluc, compared with the crRNA control, even in cells that did not express the specific protein (Fig. 4a), suggesting that the riboswitch approach may not have been able to completely block crRNA activity. To solve this problem, we simultaneously inserted the lncRNA functional motif into both the 5′- and 3′-ends of the crRNA. In this manner, the riboswitch at the 3′-end was used to bind the endogenous protein and open the crRNA, and the binding motif at the 5′-end was used to recruit the endogenous transcription regulation system (Fig. 4b). After adopting this approach, we found that neither inducible CRISPR transcriptional activation nor inhibition system showed any leakage (Fig. 4c). As the endogenous protein level increased, these inducible CRISPR systems also showed a broader signal response range than previous systems (Fig. 4d). Taken together, these results suggested that simultaneous inducible control of transcription after modification at both ends of crRNA solved the problems of systemic leakage and insensitive signaling responses in the previous inducible CRISPR transcription regulation system.

### Inhibition of CRISPR transcriptional activity based on endogenous signals

**Fig. 5:**
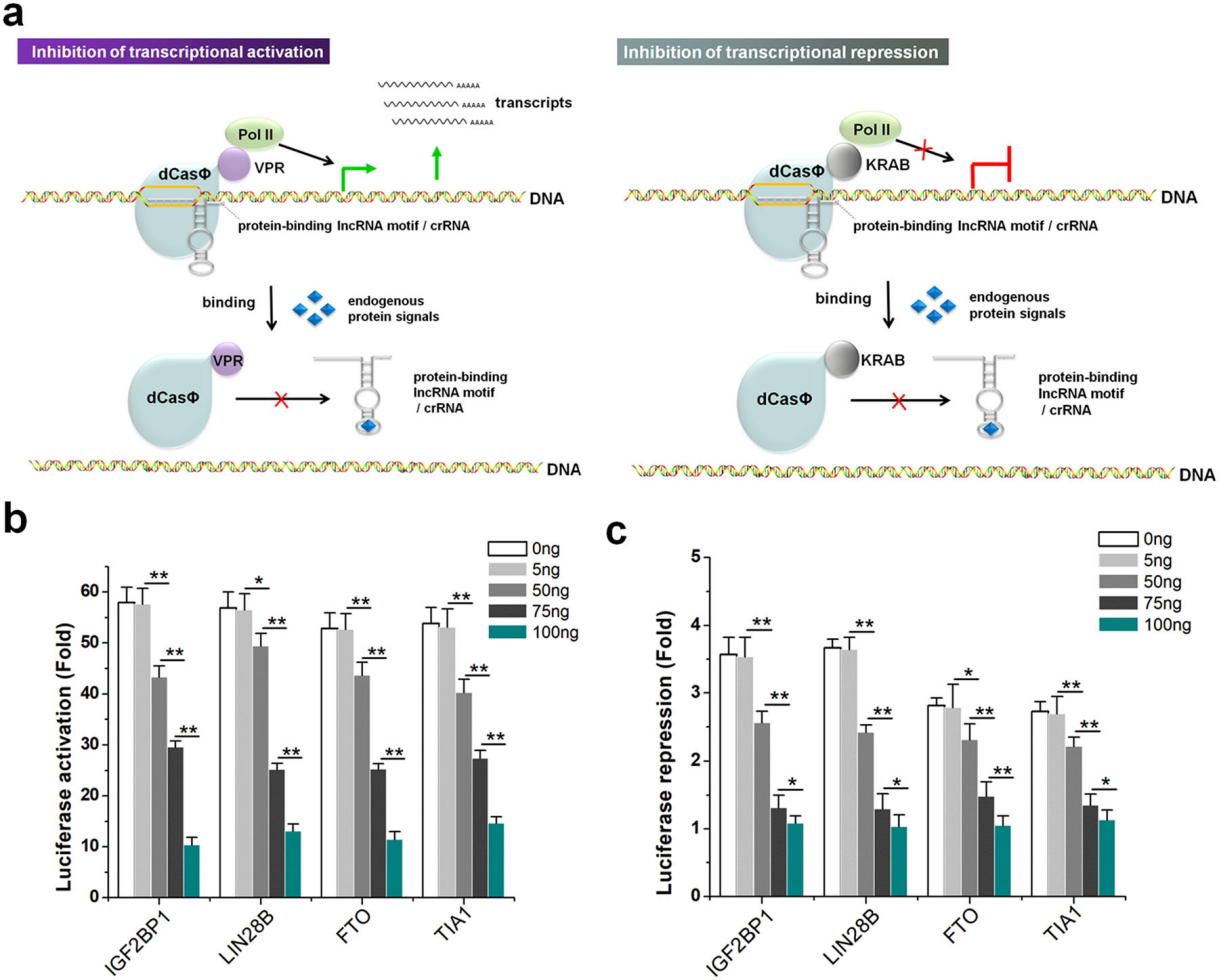
Inhibition of CRISPR transcriptional activity by endogenous protein signals. **a** Schematic diagram showing the inhibition of CRISPR transcriptional activity by endogenous protein signals. **b** The transcriptional regulatory activity of dCasΦ-VPR decreased significantly with the increase in the amount of transfected overexpression plasmid. **c** The transcriptional regulatory activity of dCasΦ-KRAB decreased significantly with increases in the amount of transfected overexpression plasmid. Relative luciferase activities were determined as the ratios of Rluc to Fluc values. The fold change in each group was determined by comparison with the wild-type crRNA negative control. The reported data represent the means ± SD from five independent experiments. *P < 0.05; **P < 0.01, relative to the control, two-tailed *t*-test.

Next, we inserted the lncRNA motifs that bind to cellular proteins into the middle stem-loop of crRNA to test the effect of endogenous protein on dCasΦ-VPR and dCasΦ-KRAB system activities (Fig. 5a). Previous studies have suggested that inserting an aptamer into the middle stem-loop of a sgRNA in the CRISPR-dCas9 system might not affect the transcriptional regulatory activity of the CRISPR system^38^. However, 48 h after transfection, the luciferase assay in HEK-293 cells indicated that the transcriptional regulatory activities of the dCasΦ-VPR (Fig. 5b) and dCasΦ-KRAB (Fig. 5c) systems both decreased significantly with increases in the amount of transfected overexpression plasmids, suggesting that the binding of lncRNA elements to endogenous proteins may have inhibited the binding of the dCasΦ protein to crRNA (Fig. 5a). Correspondingly, as the concentration of shRNAs increased, we observed the opposite trend (Supplementary Fig. S8). These results suggested that endogenous proteins can be used to inhibit crRNA activity when the lncRNA protein-binding sequence is inserted into the middle stem-loop.

### Construction of multi-input logic gates using CRISPR signal conductor 2.0

**Fig. 6:**
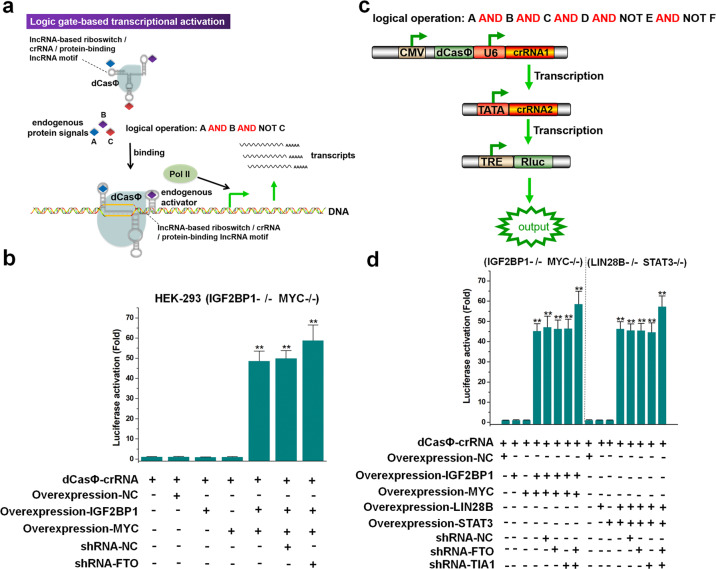
Design and construction of multi-input logic gates using signal conductor 2.0. **a** Schematic diagram showing the logic gate-based transcriptional activation. The CRISPR-dCasΦ system activated the expression of target gene only when the logical condition “A AND B AND NOT C” was established. **b** The expression level of Rluc was regulated by the logic gate, which performed the “LIN28B AND STAT3 AND NOT TIA1” operation. **c** Schematic diagram showing the logic gate performing the logical operation “A AND B AND C AND D AND NOT E AND NOT F.” **d** The expression level of Rluc was regulated by the logic gate, which performed the “IGF2BP1 AND MYC AND LIN28B AND STAT3 AND NOT FTO AND NOT TIA1” operation. Relative luciferase activities were determined as the ratios of Rluc to Fluc values. The fold change in each group was determined by comparison with the crRNA negative control. Error bars represent the SD from five independent experiments. *P < 0.05; **P < 0.01, relative to the control, two-tailed *t*-test.

The high compatibility and controllability of the crRNA in the dCasΦ system prompted us to construct multi-input logic gates to demonstrate the information integration capabilities of CRISPR signal conductor 2.0. First, we designed a logic gate that performed “A AND B AND NOT C” logical operations (Fig. 6a). We inserted the riboswitch that senses the IGF2BP1 protein, the lncRNA motif that recruits the MYC protein, and the lncRNA motif that binds to the FTO protein into the 3′-end, 5′-end, and the middle loop regions of crRNA, respectively. We first validated the system in HEK-293 cells that stably expressed TRE-Rluc and in which both alleles of *IGF2BP1* and *MYC* genes were deleted. No significant expression of Rluc was observed 48 h after transfection of dCasΦ (Fig. 6b). However, the expression of Rluc was significantly increased (Fig. 6b) when IGF2BP1 and MYC overexpression vectors were co-transfected at the same time. The Rluc expression was not detected after co-transfection of one of these two vectors. We then co-transfected shRNA-FTO with the overexpression vectors into cells and found that the expression of Rluc was further improved to a certain extent (Fig. 6b). To demonstrate the universality of this strategy, we constructed another logic gate that performed the “LIN28B AND STAT3 AND NOT TIA1” operation. By knocking down TIA1 and overexpressing LIN28B/STAT3 in LIN28B/STAT3-double-knockout HEK-293-TRE-Rluc cells, we observed similar results (Supplementary Fig. S9). To further enhance the integration of input signals, we connected the two aforementioned logic gates to construct a gate that performed the “IGF2BP1 AND MYC AND LIN28B AND STAT3 AND NOT FTO AND NOT TIA1” operation, with one crRNA activating the expression of the subsequent crRNA (Fig. 6c). Through a series of combined experiments with protein overexpression and RNA knockdown in IGF2BP1/MYC or LIN28B/STAT3-double-knockout HEK-293-TRE-Rluc cells, we confirmed that the six-input logic gate activated Rluc expression under the abovementioned logical conditions (Fig. 6d). Together, these results indicated that these devices can logically control multiple cellular signals to produce a desired output.

### Specific recognition and inhibition in cancer cells

**Fig. 7:**
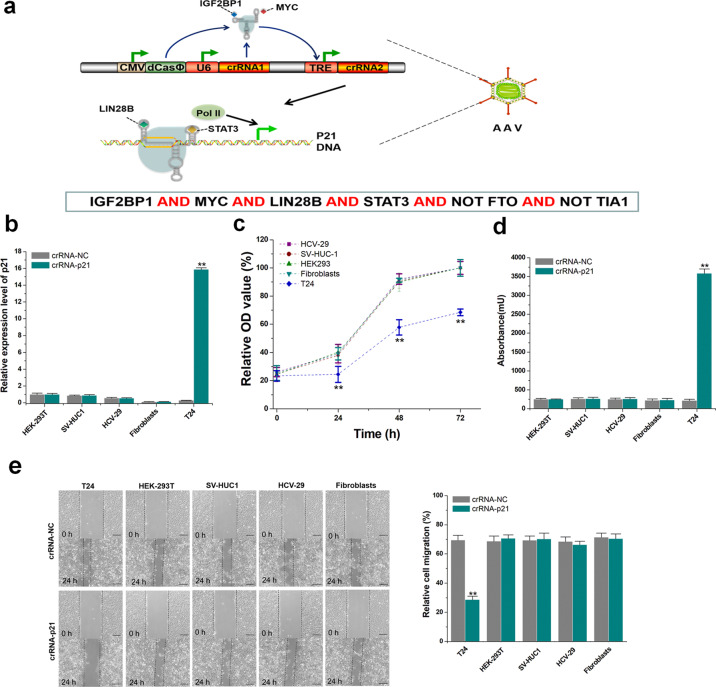
Specific recognition and inhibition of cancer cells through the AAV-logic gate. **a** Schematic diagram of the six-input logic gate delivered by AAV. **b** Changes in the relative expression levels of p21 in cancer and normal cells. The relative expression levels of p21 were determined by qPCR 48 h after AAV transduction. **c** Proliferation of transduced cells was determined by CCK-8 assay at different time points. Relative optical density (OD) values (%) in each group were determined by comparison with the crRNA negative control. **d** The level of cell apoptosis was calculated by performing the Cell Death Detection ELISA assay. **e** Migration of transduced cells was measured by wound healing assay. Relative cell migration (%) in each group was determined by the migration distance normalized to that of the crRNA negative control group. The reported data represent the means ± SD from five independent experiments. *P < 0.05; **P < 0.01 between the groups using a two-tailed *t*-test.

In preliminary studies, we found that the expression levels of the classic proto-oncogenes, *IGF2BP1*, *MYC*, *LIN28B* and *STAT3* in bladder cancer T24 cells were higher than those in normal cells, while the expression levels of the tumor suppressor genes *FTO* and *TIA1* were negligible in T24 cells (Supplementary Fig. S10). We then chose bladder cancer cells to construct a test model for use with the aforementioned 6-input logic gate constructed for specific recognition of cancer cells. The output was swapped from the luciferase gene to the endogenous tumor suppressor p21 gene, and the circuit was inserted into an all-in-one AAV2 delivery vector, which mediated the delivery of minigene circuits in bladder cancer cells as shown in our previous study (Fig. 7a). We found that transduction with the generated virus selectively increased p21 expression in bladder cancer T24 cells, but had no effect on normal cells, including two bladder transitional epithelial cell lines (HCV-29 and SV-HUC-1 cell lines), HEK-293 cells, and fibroblasts (Fig. 7b). For comparison, we performed a similar test with two logic gates that sensed only three signals. Although both gate systems were tumor-specific, the six-input logic gate showed better performance (Supplementary Fig. S11). The cell proliferation rate was then determined at various time points by Cell Counting Kit-8 (CCK-8) assay. As shown in Fig. 7c, the logic gate specifically suppressed the proliferation of the bladder cancer cells. Consistent with these findings, the logic gate also specifically induced T24 cell apoptosis, as determined by ELISA (Fig. 7d). In addition, a wound healing assay indicated that the logic gate attenuated the migration of bladder cancer cells, while normal cells were not affected (Fig. 7e). These results indicated that the six-input logic gate can be used to specifically and strongly inhibit the malignant phenotype of cancer cells.

### Activation of the inflammatory immune system by AAV-dCasΦ

**Fig. 8:**
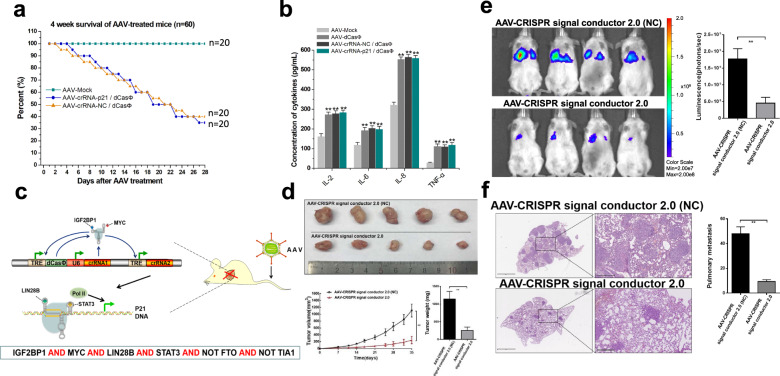
Inhibition of tumor growth and metastasis in vivo by AAV-dCasΦ. **a** The survival curve of mice showed that the tail vein injection of AAV-dCasΦ significantly shortened the survival time of the mice. **b** The ELISA method was used to detect the expression changes in immune-inflammatory indicators in the peripheral blood of mice after tail vein injection of AAV-dCasΦ. **c** Schematic diagram showing the six-input logic gate controlled by CRISPReader. **d** AAV-CRISPR signal conductor 2.0 efficiently inhibited in vivo tumor growth. The tumor volume and weight were measured at the indicated time points after cell transplantation into mice. The in vivo growth of tumors treated with the AAV-CRISPR signal conductor 2.0 was dramatically slower than that of tumors treated with the negative control using non-targeting crRNA. **e** Quantification of bioluminescence images of a metastatic model. The luminescence signal intensities are shown. **f** Histopathological inspection of mouse lungs treated with AAVs. Lungs were examined with H&E staining, and lung sections from the mice injected with different AAVs were analyzed 4 weeks after treatment. Pulmonary metastases of various sizes were observed. The data are shown as the means ± SD. *P < 0.05; **P < 0.01, between the groups, two-tailed *t*-test.

The specific and highly effective anti-cancer ability of AAV-dCasΦ in cell lines motivated us to test it in animal models. However, in preliminary experiments, we unexpectedly found that the injection of AAV-dCasΦ through the tail vein caused the death of wild-type mice. Both the crRNA-p21/dCasΦ and the crRNA-negative control/dCasΦ treatments caused the decline of mouse survival within 4 weeks (Fig. 8a), but the injection of the same dose of AAV that did not express dCasΦ did not cause this effect, thus suggesting that dCasΦ may cause an immune-inflammatory response. To confirm this possibility, we performed ELISA to detect the expression of the immune-inflammatory factors IL-2, IL-6, IL-8, and TNF-α in the peripheral blood of mice 1 week after AAV injection. The results showed that the expression of these four inflammatory indicators was significantly increased in the AAV-dCasΦ group (Fig. 8b), indicating that high expression of dCasΦ protein in the body may cause certain inflammatory side effects.

### Inhibition of tumor growth and metastasis in vivo

We hypothesized that the abnormal activation of the immune-inflammatory system may be attenuated by conditional control of dCasΦ expression in cancer cells. Using CRISPReader technology that we had developed previously^33^, we tried a new way of initiating and regulating gene expression using the six-input logic gate. As shown in Fig. 8c, crRNA1 was designed to bind to the TRE promoter, which then drove the expression of dCasΦ and crRNA2. After initial expression, crRNA1/dCasΦ then bound the upstream TRE promoter and amplified dCasΦ expression through a positive feedback mechanism. Then, dCasΦ/crRNA1 further activated the expression of crRNA2, which in turn activated the expression of the p21 gene. The logical conditions needed to be properly set for crRNA to function in this manner. dCasΦ can accumulate in large amounts only in cells highly expressing IGF2BP1 and MYC, but do not express FTO. Furthermore, the cellular p21 gene can only be activated when the aforementioned six-input conditions are simultaneously met. To test the true effect of the six-input logic gate, we injected the AAV-mediated construct into the tail vein of wild-type mice and then monitored the survival of the mice and changes in the expression of immune inflammation indicators. The results indicated that this system did not show significant side effects on mice during at least 4 weeks of observation, and there was no significant change in the levels of the four immune-inflammatory indicators (Supplementary Fig. S12). To further identify potential applications, we conducted in vivo experiments with subcutaneous tumor models and found that the volume and weight of xenograft tumors were decreased by AAV-CRISPR signal conductor 2.0 compared with those in the non-targeting control group (Fig. 8d). The T24 cell line stably expressing luciferase was then used to develop an in vivo bladder cancer lung metastasis model. After injecting AAVs through the tail vein, this device significantly suppressed lung metastases (Fig. 8e). Hematoxylin and eosin (H&E) staining was then performed on the lung tissue to observe any metastasis in the groups. AAV-CRISPR signal conductor 2.0 significantly reduced the number and size of pulmonary metastases (Fig. 8f). To further investigate the specificity of this device in vivo, the dCasΦ and p21 expression levels in normal tissues were also determined. No significant expression changes in these two genes were found in normal tissues (Supplementary Fig. S13). These results suggested that AAV-mediated CRISPR signal conductor 2.0 can be used specifically to treat cancer cells in an efficient and safe manner.

## Discussion

Understanding the overall changes in cancer at the molecular level is key to understanding cancer evolutionary mechanisms and developing precise treatments. Previous genome and transcriptome studies of different malignant cancers have shown that cancers are polygenic diseases^50^, and that there is often no single specific target in any cancer. Transcription factors, RNA-binding proteins, miRNAs, and other factors show changes in expression or abnormal activities in cancer cells. Therefore, precise treatment of cancer requires simultaneous recognition of multiple and different signals. On the basis of comprehensively identifying and analyzing multiple input signals, artificial gene circuits represented by logical gates can specifically recognize and distinguish a particular cellular state and show good prospects for effectively treating human diseases^9,11–13^. There have been attempts to use AND gate-based gene circuits to specifically target and recognize cancer cells in synthetic biology^51,52^. In contrast, the strategy of overexpressing a single therapeutic gene used in traditional gene therapy does not lead to the integration of multiple protein signals, nor can it directly rewire endogenous signaling pathways in host cells. Furthermore, the expression of an introduced exogenous tumor suppressor gene may be inhibited by many host factors, often failing to exert the ideal anti-cancer effect.

A key obstacle to the creation of artificial gene circuits is the lack of programmable, scalable, and highly targeted gene regulatory tools. The RNA-mediated CRISPR system offers a potentially effective tool for building gene circuits due to its potential to edit gene sequences and regulate gene expression^20–23^. An important principle for constructing complex gene regulatory circuits is based on the use of output signals from upstream components as the input signals to downstream components. CRISPR signal conductor version 1.0^24^ uses an elongated sgRNA with a signal-dependent riboswitch. These two seemingly unrelated molecules were combined to form an RNA scaffold that was used to sense input signals such as NF-κB and β-catenin protein signals, which are highly expressed in cancer cells, to control the release of the spacer sequence of a sgRNA and regulate the transcription of downstream target genes. This method uses intracellular signals for quantitatively regulating CRISPR system activity and effectively inhibits multiple malignant biological behaviors of cancers by manipulating the direction of intracellular signal flows in cancer cells. Due to the large coding size of traditional complex gene circuits and the difficulty of integrating them into AAV vectors with clinical applications, we developed CRISPReader technology^33^ to control the expression of promoter-less genes in a robust way and simplify the CRISPR gene expression frame. Then, based on CRISPReader, we constructed a minigene circuit delivered by a single AAV^52^ and demonstrated its ability to selectively identify and effectively treat bladder cancer both in vitro and in vivo. Although this CRISPR-based gene circuit has been demonstrated to treat malignant tumors, further improving the sensing range and processing of different cellular signals has always been a key goal due to the heterogeneity of tumors, and this goal remains to be realized.

Because of their diverse regulatory effects and important functions, lncRNAs that contain multiple key functional motifs are expected to be used to create the largest target library for the development of gene therapy drugs^34–36,53^. However, the use of full-length lncRNA molecules for gene therapy causes obvious side effects^53^. In addition, RNAs can be unstable and easily degraded, and it is difficult to accurately locate specific targets. Inserting shorter lncRNA functional elements into a sgRNA may effectively leverage the Cas/sgRNA complex to solve the stability problem associated with noncoding RNAs and recruit them to target sequences by using the positioning ability of the CRISPR system. Moreover, the CRISPR system can be used to identify lncRNA functional motifs because the CRISPR system cannot play a regulatory role when the motifs are non-functional.

In this study, we integrated lncRNA functional motifs that bind transcription factors or other types of RNA-binding proteins into different parts of the sophisticated crRNA of dCasΦ. The functional elements at the 5′-end of crRNA recruit endogenous transcriptional regulatory factors by binding the transcription factors ATF3, MYC, STAT3, YBX1, EZH2, PRC2, LSD1, or DNMT1, thereby exerting powerful transcriptional regulatory activities without the need for classic transcriptional regulatory factors such as VPR or KRAB. The functional element in the 3′-end of crRNA blocks the DNA-binding sequence of crRNA through the regulatory riboswitch, and then reactivates crRNA in the presence of corresponding endogenous protein signals, including the abovementioned transcription factors and other proteins such as IGF2BP1, LIN28B, FTO, and TIA1. When both the 5′- and 3′-ends of crRNA were bound to the same transcription factor, crRNA showed more sensitive signal sensing ability than the previous CRISPR signal conductor, without showing any leakage. When the lncRNA functional motif was inserted into the intermediate region of crRNA, the ability of crRNA to bind dCasΦ protein was inhibited in the presence of the target protein signal, and thus the endogenous protein significantly shut down the function of crRNA. Modified crRNA can be used to construct a logic gate that simultaneously responds to three inputs, and by connecting two crRNAs in series, it can also perform logical operations based on six inputs. Prior to this study, the logic AND gate usually sensed only two input protein signals, which had corresponding RNA aptamers^24,54^. On the basis of the outstanding control ability observed in this study, we named the designed reprogrammed dCasΦ device CRISPR signal conductor version 2.0.

To further investigate the potential of CRISPR signal conductor 2.0 in disease treatment, we then used an all-in-one AAV vector to deliver the system and chose bladder cancer as the model for gene therapy studies. CRISPR signal conductor 2.0 accurately identified bladder cancer cells based on sensing and analyzing six-protein signals in the cells, and further inhibited cancer cell proliferation/migration and induced apoptosis without affecting normal cells. We also horizontally compared the system that sensed six signals and the control system that sensed only three signals and found that the system with six-input signals showed advantages in specifically targeting bladder cancer cells. In contrast, CRISPR signal conductor 1.0 did not sense these protein signals nor could it be delivered by a single AAV.

When we injected AAV-CRISPR signal conductor 2.0, specifically, AAV-dCasΦ, into the tail vein of mice with bladder cancer in vivo to perform gene therapy, we unexpectedly found that the survival time of the mice was significantly shortened. We measured immune-inflammatory indicators in the blood and found that the levels of IL-2, IL-6, IL-8, and TNF-α were significantly increased, indicating an abnormal immune response. The relationship between the occurrence of this event and the widespread expression of dCasΦ still needs to be further explored.

To specifically express dCasΦ in cancer cells, we originally planned to use cancer-specific promoters. However, these promoters may have certain transcriptional activity in some normal cells undergoing active proliferation, and some promoters show weak activity in cancer cells^51^. Therefore, to continuously express dCasΦ in cancer cells and take advantage of the precision of the six-input signal logic gate, we used CRISPReader technology to achieve specific and high expression of dCasΦ in bladder cancer cells in vivo, while inducing no dCasΦ expression in normal cells. Notably, the survival time and immune factor expression in the mice treated with this system were not significantly affected, and this strategy inhibited the growth of subcutaneously transplanted tumors in nude mice and also suppressed the lung metastases of bladder cancer. Therefore, this approach showed effectiveness and biosafety in cancer treatment.

Although we tried to integrate relatively short functional lncRNA motifs into the crRNA scaffold of CRISPR-CasΦ, it will be interesting to determine how big the insertion of noncoding RNA motifs can be tolerated in crRNA at different positions in the near future. Adding a lncRNA protein-binding motif to the middle of crRNA led to analog regulation not digital-like switching, suggesting that this strategy may need to be further optimized to be more suitable for building logic gates.

In conclusion, this study initially demonstrated the effect of integrating lncRNA functional motifs into a CRISPR signal conductor system. In the future, it will be necessary to study and identify additional lncRNA functional elements so that the system can treat more tumors, as well as rare genetic diseases.

## Materials and methods

### Cell culture and gene transfection

The HEK-293T, T24, and SV-HUC1 cell lines used in this study were purchased from the American Type Culture Collection (Manassas, VA, USA). The HCV-29 cell line was purchased from Biotool Biological Technology (Shanghai, China). Normal human primary fibroblasts derived from the epidermis were kindly provided by Dr. T. Chen (Shantou University, Shantou, China). All cell lines were maintained in Dulbecco’s Modified Eagle’s Medium supplemented with 10% fetal bovine serum (Invitrogen, Carlsbad, CA, USA) in the presence of 5% CO_2_ at 37 °C in an incubator. For transient transfection experiments, the cells that reached 70%–80% confluency were transfected with the constructed plasmids using Lipofectamine 3000 (Invitrogen) according to the manufacturer’s protocols.

### Plasmid construction

The plasmid vector pPP441 (plasmid #158801; Addgene, Watertown, MA, USA) was used to transiently express human codon-optimized CasΦ protein in mammalian cells. Mutations in the CasΦ gene were introduced by GG-assembly to create the dCasΦ gene. The dCasΦ-VPR-crRNA and dCasΦ-KRAB-crRNA constructs were assembled by fusing dCasΦ with VPR and KRAB, respectively, to the C-terminus. All sgRNAs and derivatives were designed, chemically synthesized, and inserted into the same backbone vector digested with restriction endonucleases. All vectors were transformed into One Shot TOP10 Chemically Competent *E. coli* cells. The desired expression clones were identified using PCR amplification and electrophoresis, and then confirmed by Sanger sequencing. The sequence information of the related elements is shown in Supplementary Table S1.

### Relative luciferase assay

Both the Rluc and Fluc activities were measured in a 1.5-mL Eppendorf tube using a Promega Dual-Luciferase Reporter Assay Kit (E1980; Promega, Madison, WI, USA) according to the manufacturer’s protocols 48 h after DNA transfection. The relative luciferase activity was calculated as the Rluc value normalized to the Fluc value. The assays were performed in triplicate and experiments were repeated five times.

### RNA extraction and RT-qPCR

TRIzol reagent (Invitrogen) was used to extract total RNA from cells transfected with the plasmids and mouse tissues according to the manufacturer’s protocols. Total RNA was reverse transcribed to cDNA using a RevertAid^TM^ First Strand cDNA Synthesis Kit (Fermentas, Hanover, MD, USA) according to the related protocols. The RT-qPCR reactions were performed on an ABI PRISM 7000 Fluorescent Quantitative PCR System (Applied Biosystems, Foster City, CA, USA) using an All-in-One™ qPCR Mix (GeneCopoiea, Rockville, MD, USA). The PCR cycling parameters were as follows: 95 °C for 15 min, followed by 40 cycles of 94 °C for 15 s, 55 °C for 30 s, and 72 °C for 30 s. Glyceraldehyde 3-phosphate dehydrogenase (GAPDH) was used as the internal control, and the data were normalized to the expression of GAPDH. Relative gene expression was calculated using the Delta-Delta-Ct (^ΔΔ^Ct) algorithm. The reported values represent the means ± SD of five biological replicates. The sequence information of the related primers is shown in Supplementary Table S2.

### Cell proliferation assay

Cell proliferation was monitored using a CCK-8 Assay Kit (TaKaRa, Kusatsu, Japan) according to the manufacturer’s protocols. Cells were seeded in a 96-well plate for 24 h, and transiently transfected with the plasmids. Then, 0, 24, 48, or 72 h post transfection, 10 µL of CCK-8 reagent was added to each well and the cells were incubated for 1 h. Absorbance was measured at 450 nm using a microplate reader (Bio-Rad, Hercules, CA, USA). The assays were independently repeated five times.

### Cell migration assay

Cell migration was determined by a wound healing assay. Briefly, cells were seeded in 12-well plates at equal densities and grown to 90% confluence. A clean scratch was created by using a sterile 200 μL pipette tip to make a scratch on the monolayer 5 h post transfection. Areas of wounding were marked and photographed with a digital camera at 0 h and 20 h after injury. The cell migration distance (mm) was calculated using HMIAS-2000 software. Each experiment was repeated five times.

### Cell apoptosis assay

Cell apoptosis was detected by calculating histone-complexed DNA fragments (nucleosomes) in the cytoplasm with a cell death detection ELISA kit (Roche Applied Science, Penzberg, Germany) according to the protocols. The absorbance was measured at 405 nm using a microplate reader (Bio-Rad) and the value was considered to be proportional to the amount of nucleosomes released into the cytoplasm. Each assay was repeated five times.

### AAV packaging, purification, and titer detection

The pAAV packaging plasmid, pHelper plasmid, and a pAAV plasmid were co-transfected into HEK-293T cells using Lipofectamine 3000 (Invitrogen). The culture supernatants were collected 48 h after plasmid transfection, concentrated, and used as virus stocks for subsequent AAV infection experiments. The AAV titer was calculated by qPCR using 2× EvaGreen Master Mix (Syngentech, Shanghai, China).

### Tumor xenografts

The procedure for the tumor xenograft assay was approved by the Shenzhen University Ethics Committee. The mice were housed under standard laboratory conditions. BALB/c-nude mice were randomly assigned into either the experimental group or control groups (five mice for each group). Bladder cancer T24 cells (5 × 10^7^) were hypodermically injected into the backs of BALB/c-nude mice, and then, a single injection of AAV (100 μL, 2 × 10^11^ vp/mL) via the tail vein was conducted 10 days after initial inoculation. Next, tumor volumes were calculated using the formula: V = L × W^2^/2, where L is the length and W is the width of the tumor. The mice were sacrificed and tumors were removed at the end of the experiment.

### Experimental metastatic mouse model

T24 bladder cancer cells stably expressing luciferase (1 × 10^5^ cells) were suspended in 200 μL of phosphate-buffered saline and injected into the lateral tail vein of 5-week-old male B-NDG mice (Biocytogen, Beijing, China). Four weeks later, the mice were anaesthetized with isoflurane, and D-luciferin sodium salt (150 mg/kg) was injected intraperitoneally. Bladder cancer cells were counted with an in vivo imaging system, Xenogen IVIS (PerkinElmer, Boston, MA, USA). The total flux of photons per second was calculated for the lung region using Living Image 4.3.1 software (PerkinElmer/Caliper).

### Quantification of IL-2, IL-6, IL-8, and TNF-α levels

BALB/c mice were injected with AAV (100 μL, 2 × 10^11^ vp/mL) in the tail vein. IL-2, IL-6, IL-8, and TNF-α levels in the peripheral blood of the mice were detected with commercially available ELISA kits (AlerCHEK, Portland, ME, USA) according to the manufacturer’s guidelines. Detection was performed with a microplate reader (Bio-Rad), and a standard curve was drawn based on the results. The concentration of each cytokine was then calculated.

### H&E staining of lung tissues

Mouse lung tissues were fixed in 10% formalin and dehydrated in ethanol. Paraffin embedding, sectioning, and H&E staining were performed according to the manufacturer’s procedures, and then slides were imaged with a Nikon Ci-L bright field microscope.

### Statistical analyses

No statistical methods were used to predetermine the sample size. The investigators were blinded to allocations during the experiments and outcome assessments. Statistical analysis was conducted using the *t*-test or analysis of variance, and *P* < 0.05 was considered statistically significant. All statistical tests were performed using SPSS statistical software for Windows version 20.0 (SPSS, Chicago, IL, USA).

## Supplementary information

Supplementary information

## Data Availability

The data generated or analyzed during this study are included in this paper and its supplementary materials. The data supporting the findings in the main text are found in Supplementary Figures. The sequences of elements used in this study are listed in Supplementary Tables. All data are available from the corresponding author upon reasonable request.
