# Supplementary material for: CRISPR signal conductor 2.0 for redirecting cellular information flow
Source: Cell Discov. 2022 Mar 15;8:26. doi: 10.1038/s41421-021-00371-1 (PMC8921274; doi:10.1038/s41421-021-00371-1)
Supplement: Supplementary file 1 — Supplementary information [file 41421_2021_371_MOESM1_ESM.pdf]

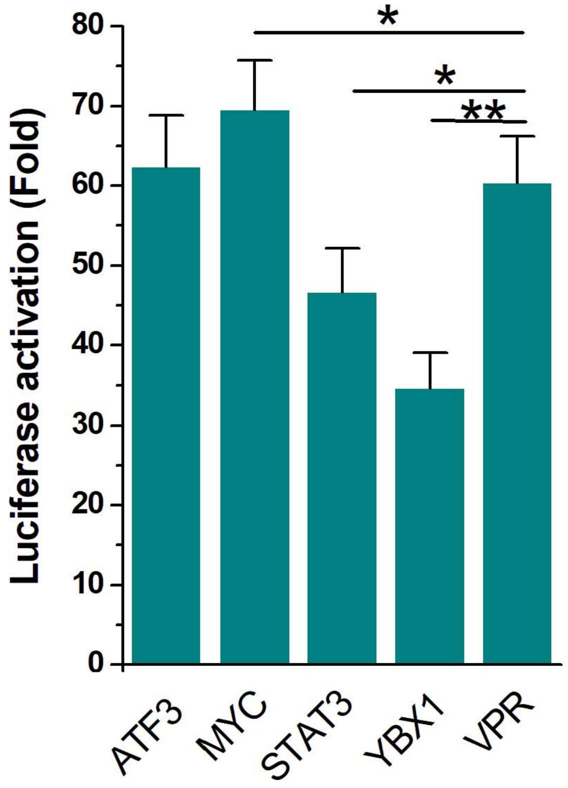

Supplementary Figure S1. The fold-activation of Renilla luciferase (Rluc) in HEK293 cells treated by various devices. The relative luciferase activity was measured at 48 h post-transfection. Each experiment was performed in triplicate for five independent times. The fold change in each group was determined by comparison with the crRNA negative control. Each error bar indicates the variation between the means of five independent experiments. *, p value < 0.05, and **, p value < 0.01, relative to the control by two-tailed *t*-test.

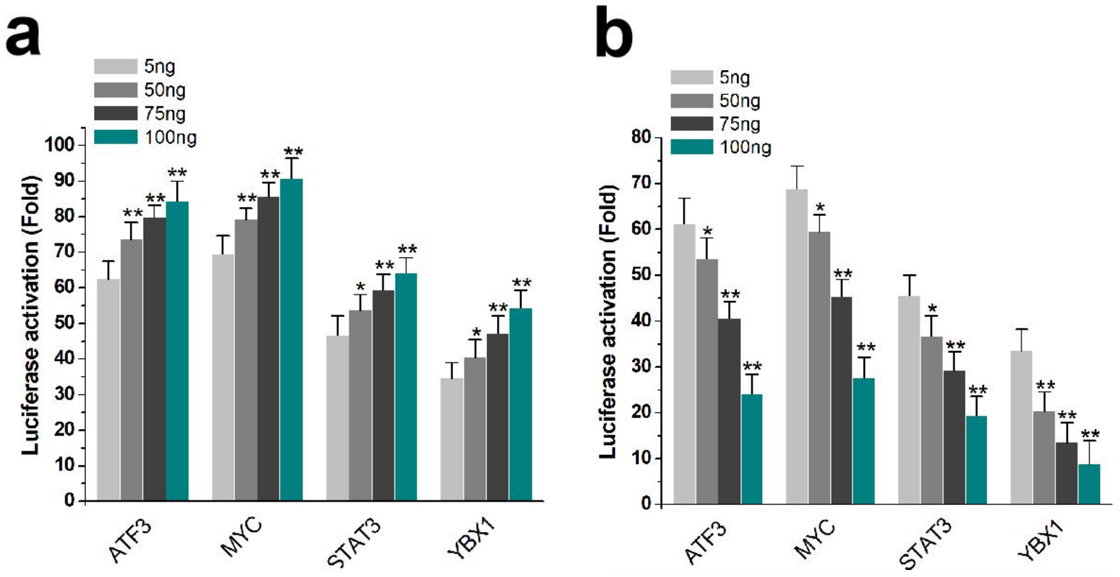

Supplementary Figure S2. The fold-activation of Renilla luciferase (Rluc) in HEK293 cells treated by plasmids of various amounts. (**a**) The fold-activation of luciferase is positively correlated with the amount of transfection of the gene overexpression plasmids. (**b**) The fold-activation of luciferase is negatively correlated with the amount of transfection of the shRNA-encoding plasmids. The relative luciferase activity was measured at 48 h post-transfection. Each experiment was performed in triplicate for five independent times. The fold change in each group was determined by comparison with the crRNA negative control. Each error bar indicates the variation between the means of five independent experiments. *, p value < 0.05, and **, p value < 0.01, relative to the control by two-tailed *t*-test.

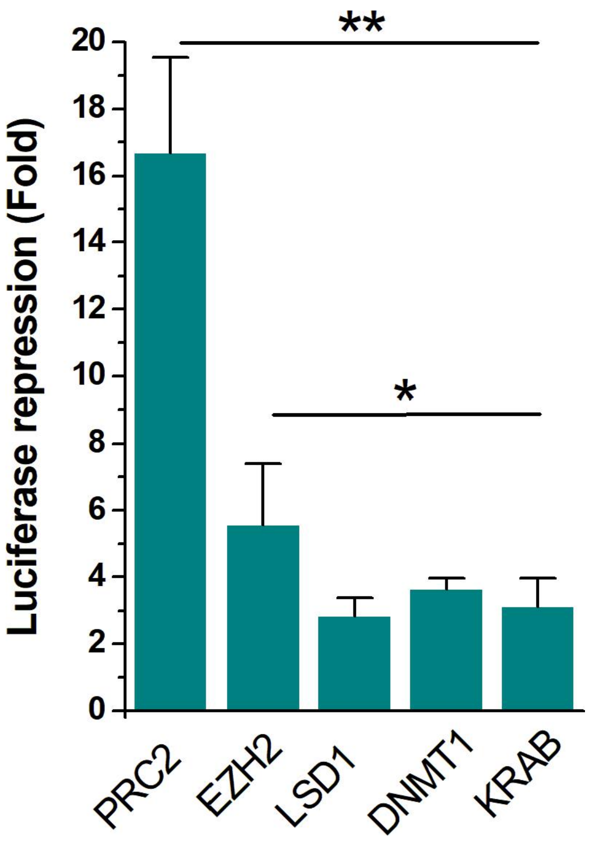

Supplementary Figure S3. The fold-repression of Renilla luciferase (Rluc) in HEK293 cells treated by various devices. The relative luciferase activity was measured at 48 h post-transfection. Each experiment was performed in triplicate for five independent times. The fold change in each group was determined by comparison with the crRNA negative control. Each error bar indicates the variation between the means of five independent experiments. *, p value < 0.05, and **, p value < 0.01, relative to the control by two-tailed *t*-test.

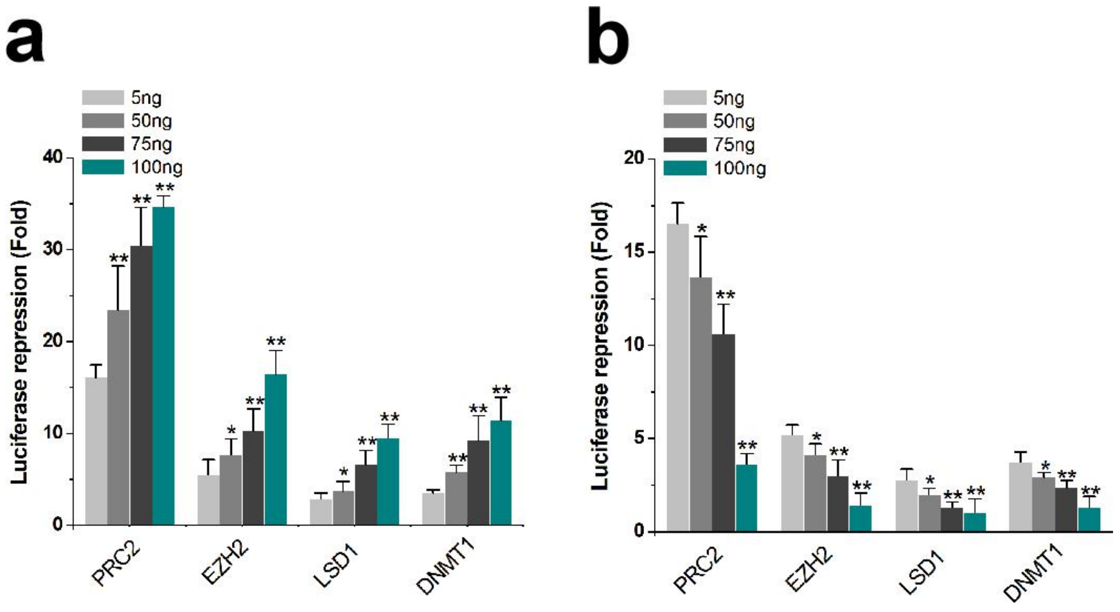

Supplementary Figure S4. The fold-repression of Renilla luciferase (Rluc) in HEK293 cells treated by plasmids of various amounts. (**a**) The fold-repression of luciferase is positively correlated with the amount of transfection of the gene overexpression plasmids. (**b**) The fold-repression of luciferase is negatively correlated with the amount of transfection of the shRNA-encoding plasmids. The relative luciferase activity was measured at 48 h post-transfection. Each experiment was performed in triplicate for five independent times. The fold change in each group was determined by comparison with the crRNA negative control. Each error bar indicates the variation between the means of five independent experiments. *, p value < 0.05, and **, p value < 0.01, relative to the control by two-tailed *t*-test.

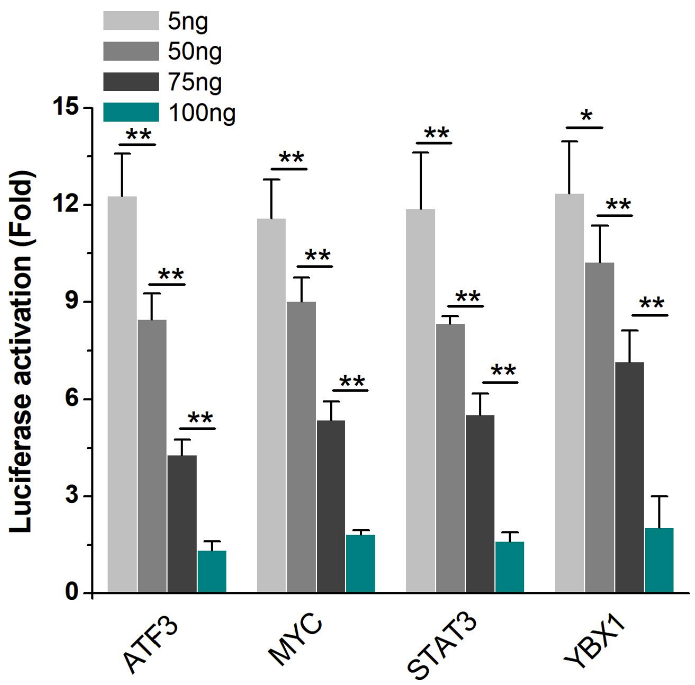

Supplementary Figure S5. The effect of RNAi on the inducible activation of Renilla luciferase (Rluc) in HEK293 cells treated by dCasΦ-VPR device. The fold-activation of luciferase is negatively correlated with the amount of transfection of the shRNA-encoding plasmids. The relative luciferase activity was measured at 48 h post-transfection. Each experiment was performed in triplicate for five independent times. The fold change in each group was determined by comparison with the crRNA negative control. Each error bar indicates the variation between the means of five independent experiments. *, p value < 0.05, and **, p value < 0.01, relative to the control by two-tailed *t*-test.

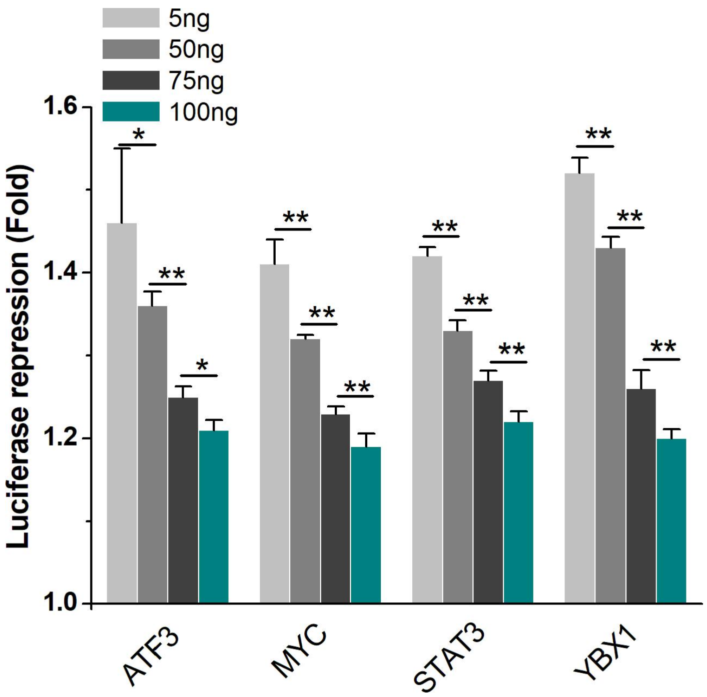

Supplementary Figure S6. The effect of RNAi on the inducible repression of Renilla luciferase (Rluc) in HEK293 cells treated by dCasΦ-KRAB device. The fold-repression of luciferase is negatively correlated with the amount of transfection of the shRNA-encoding plasmids. The relative luciferase activity was measured at 48 h post-transfection. Each experiment was performed in triplicate for five independent times. The fold change in each group was determined by comparison with the crRNA negative control. Each error bar indicates the variation between the means of five independent experiments. *, p value < 0.05, and **, p value < 0.01, relative to the control by two-tailed *t*-test.

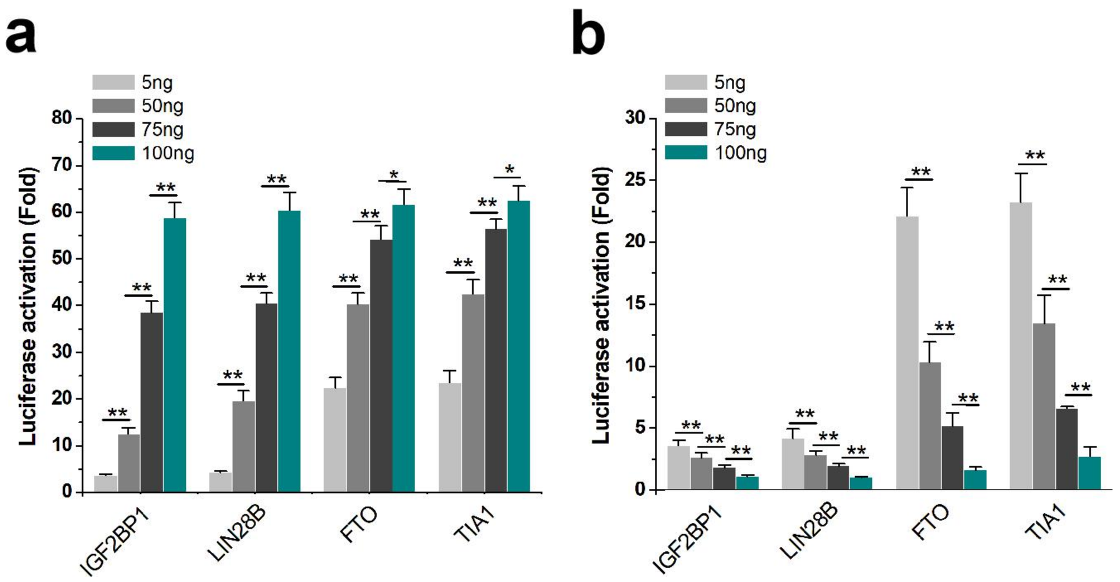

Supplementary Figure S7. The fold-activation of gene detected by dual-luciferase reporter gene assay. (**a**) The fold-activation of luciferase is positively correlated with the amount of transfection of the gene overexpression plasmids. (**b**) The fold-activation of luciferase is negatively correlated with the amount of transfection of the shRNA-encoding plasmids. The relative luciferase activity was measured at 48 h post-transfection. Each experiment was performed in triplicate for five independent times. The fold change in each group was determined by comparison with the crRNA negative control. Each error bar indicates the variation between the means of five independent experiments. *, p value < 0.05, and **, p value < 0.01, relative to the control by two-tailed *t*-test.

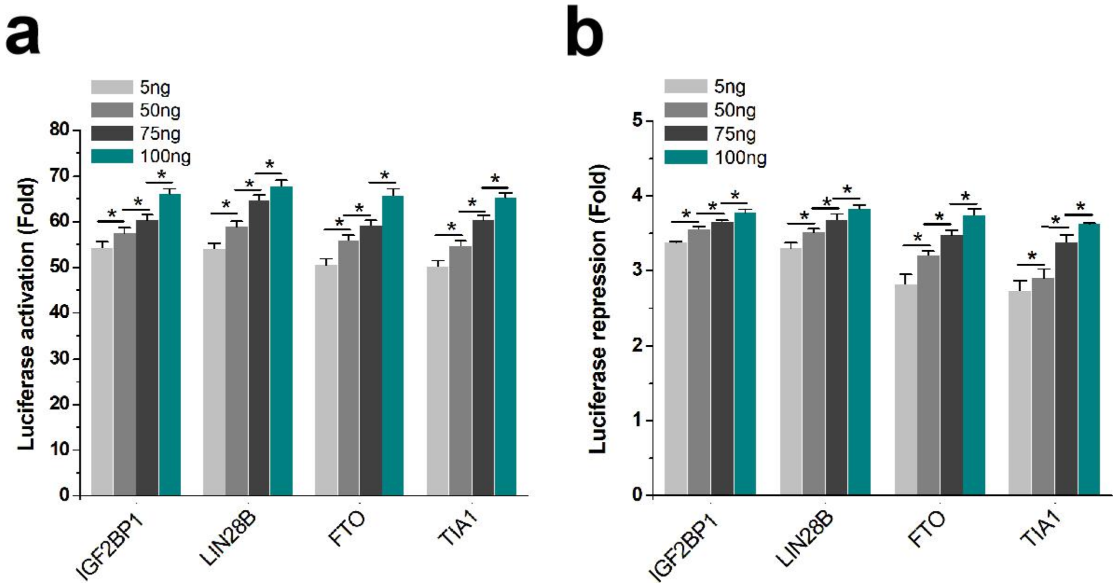

Supplementary Figure S8. The effect of RNAi on the repression of Renilla luciferase (Rluc) in HEK293 cells treated by dCasΦ-VPR (KRAB) devices. (**a**) The fold-activation of luciferase is positively correlated with the amount of transfection of the shRNA-encoding plasmids. (**b**) The fold-repression of luciferase is positively correlated with the amount of transfection of the shRNA-encoding plasmids. The relative luciferase activity was measured at 48 h post-transfection. Each experiment was performed in triplicate for five independent times. The fold change in each group was determined by comparison with the crRNA negative control. Each error bar indicates the variation between the means of five independent experiments. *, p value < 0.05, relative to the control by two-tailed *t*-test.

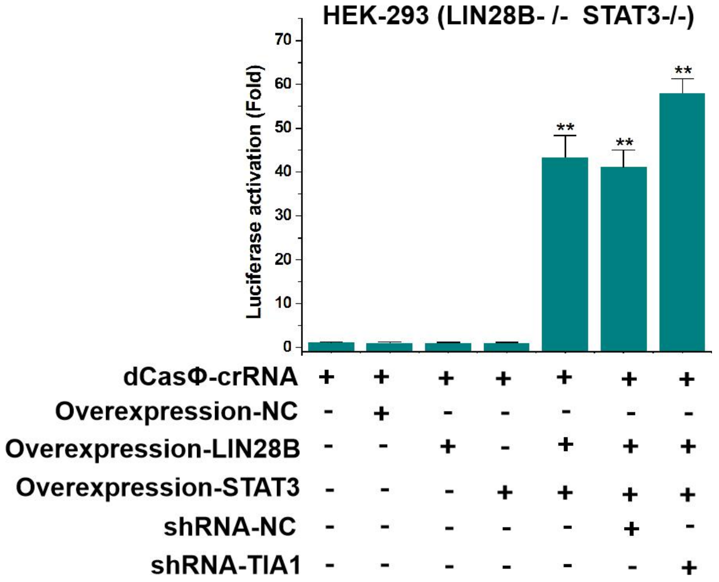

Supplementary Figure S9. The expression level of Rluc regulated by the logic gate. The logic gate performs the "LIN28B AND STAT3 AND NOT TIA1" operation. Relative luciferase activities are determined as the ratios between Rluc and Fluc values. The fold change in each group was determined by comparison with the crRNA negative control. Error bars represent the SD from five independent experiments. **, p value < 0.01, relative to the control using a two-tailed *t*-test.

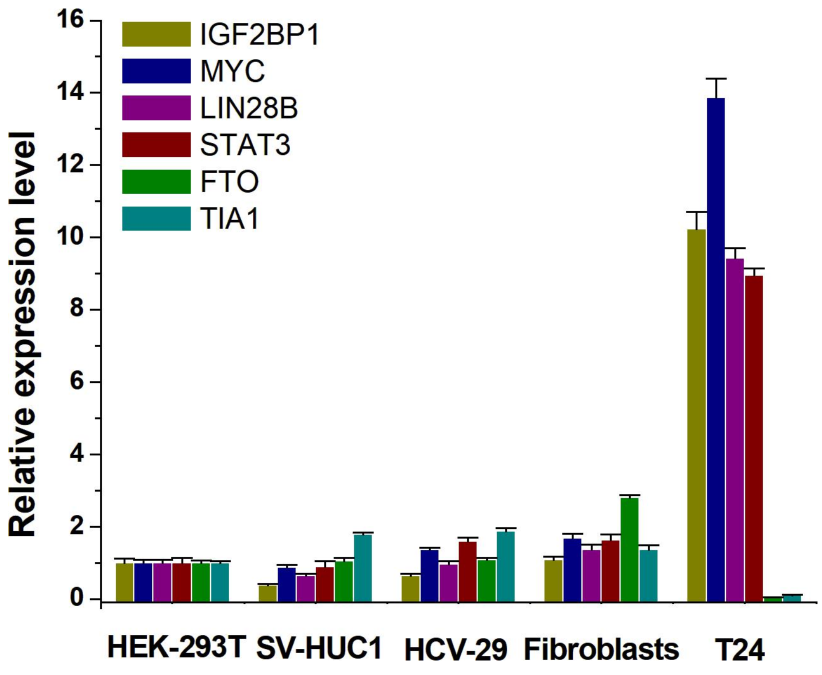

Supplementary Figure S10. The expression levels of related genes in different cell lines. The relative expression level was determined by RT-qPCR. Results are shown as the mean ± SD. Each experiment was performed in triplicate five independent times.

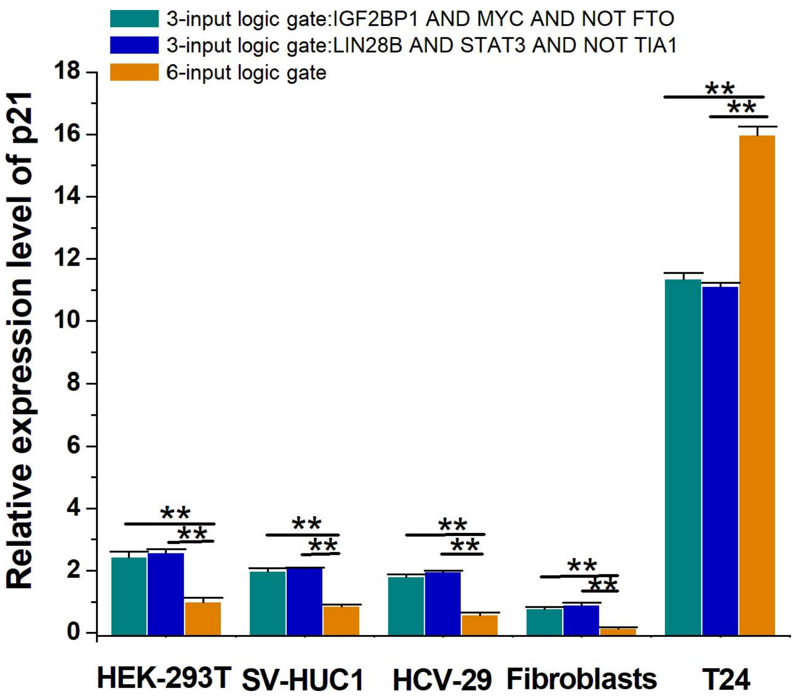

Supplementary Figure S11.The relative expression levels of p21 in cancer and normal cells. The relative mRNA expression was determined by qPCR at 48 h after transduction of AAV. Reported data are mean ± SD from five independent experiments. **p < 0.01 between the groups using a two-tailed *t*-test.

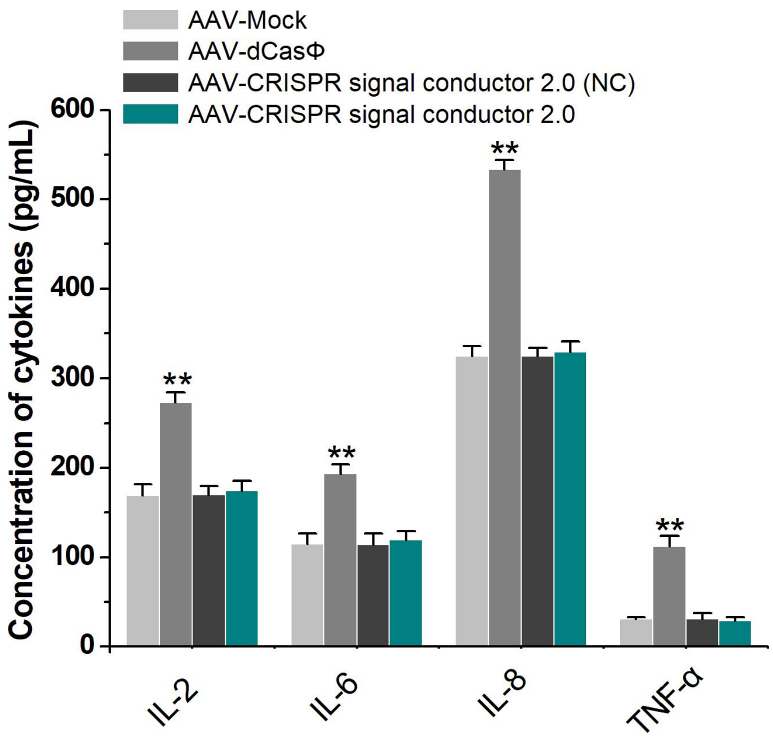

Supplementary Figure S12. Expression levels of cytokines in the peripheral blood of mice after AAV treatment. The ELISA method was used to detect the expression changes of immune inflammatory indicators in peripheral blood of mice after tail vein injection of AAVs. NC, negative control group using the non-targeting crRNA. Data are shown as the mean of mean ± SD. $^{**}p < 0.01$, between the groups, using a two-tailed *t*-test.

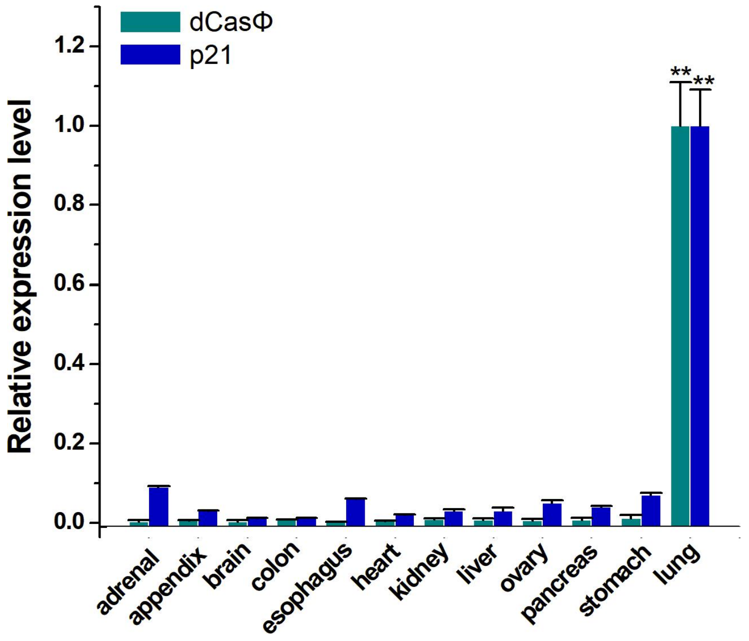

Supplementary Figure S13. Expession levels of dCasΦ and p21 in various tissues. The relative mRNA expression level was determined by qPCR. Reported data are mean ± SD from five independent experiments. **p < 0.01 between the groups using a two-tailed *t*-test.

Supplementary Table S1. The cDNA sequences of the engineered elements used in this study.

Names	Sequences
RNA-protein binding motif-IGF2BP1	CATTGAGGCAGCCAGCGCAGGGGCTTCTGCTGAGGGGGCAGGCGGAGCTTGAGGAAACCGCAGATAAGTTT
RNA-protein binding motif-LIN28B	GCCAGCGCAGGGGCTTCTGCTGAGGGGGCAGGCGGAGCTTGAGGAAACCGC
RNA-protein binding motif-FTO	GGGGACGTAGGCCGATTTCCGGGTGTTGTAGGTTTCTCTTT
RNA-protein binding motif-TIA1	TACATGGTGCTGCCAGCCCAGCTAATTA
RNA-protein binding motif-MYC	GGACGAGCCAGTTATCCCTCAGAGCTCCTGCTGCCTCGCCCGCTTTCTCTCGGAAACGTGAAGTGTGGCCTCAGCTGAAAGTGAGGTGGGCCTCATTCAATCAGTTGAATTCTTCAAGAGAGAAAAACTGAAGTCCCTTAGAAGGAAAGAGTTCT
RNA-protein binding motif-EZH2	GGAAAAGGTGGTGGAGGTAGA
RNA-protein binding motif-YBX1	ATTTGAAAATGGCCTTGATGATTCAGACGGCCGTGACGTCAGCGGGGTCAAGTTGTCGGCAGGCGGAGCGCGCAGAGTGGAGTAACAGCGCCATCTAGCAGCTGCCTCGGGGGAA
RNA-protein binding motif-PRC2	GTGGAGAGGAAAGCATTAGGGGAGCCCACGGCTACAAAAACAAGT
RNA-protein binding motif-STAT3	AGAAGTACAAAGAAAGAGAAAGTTTGGGGAGATGGATAACAAGCTCAGCTGTGTCAGTGATGTGGAGGGGAGGTATGGTGGGGGACCAGCCATGGCCCTATCCAACCCCAGGCTCCACAGGCCCCAAATTGGCTTTGCAAATCCAAACATTTTAAGGAAGTGGTTTAAGGGATGGAAGANAAACACGGTAAAAGGTCTGNGCTGTGGATTTTCATCTA
RNA-protein binding motif-ATF3	GCCCGCTGAACTCCATCCTCCCGGCGGTCGGGCGGCGGCGGCTGCGGTCGGTCGCGGCAGCGGCTC
RNA-protein binding motif-DNMT1	CCTGCTTGTCTCTGGGCTTGCACCAGCGGGTACAGACCGGAAACCTGGGCTGG

	CTCTCACTGGGTTTATTGGAGCACCTAGGCTTAGAACCTCGGATTTCTAGAACCCCGAAACCTCCGCGGTTCCCCGAACCTTAGGATCCTCTCCCACATGTCGTAGAATCTTGGAATCATGACAGCTAGAAGCGTGAAGCTCCCTCAATTCCACATACTGGGGAAAAATGAGTTGTACAAAAGGGCAGAGAGTGCAAATCTCTCTGAGCTTCAGTTTCATTTTCTACGACATGGGGATAACCCTCCGGTGCTTATAAATATTCCAGCATAACATGGCCAACCCGATGGCTCCCGAAACCTTGCCAGATGCTTCCTAGGGGAGCCAGAATTTGATCCCAACACTGATTAAAAAAAAGAAAAAAGAAAGAGAGACAGCCTCCAGGAGATTAAAGACCATGAACTTGAAGCCATA
RNA-protein binding motif-LSD1	TTAGGGTTAGGGTTAGGG
Structural sequence of riboswitch	crRNA spacer-CTCC-antisense RNA to spacer (15nt)-RNA protein binding motif-switching stem (10nt)
crRNA spacer-Rluc (TRE promoter)	TCTATCACTGATAGGGAGTA
crRNA spacer-Rluc (SV40 promoter)	TGGAATAGCTCAGAGGCCGA
crRNA spacer-MALAT1 (activation)	TAACCGGCTCTAGCCGGTCC
crRNA spacer-MALAT1 (repression)	GGACGCAGCGACGAGTTGTG
crRNA spacer-p21 (activation)	GGTGGGGCGAGTCATCGTCT
crRNA spacer-p21 (repression)	CAGGAATGCCGCAGATGTAC

Supplementary Table S2. Primer sequences used in real-time quantitative PCR.

Names	Sequences
p21-F	TGTCCGTCAGAACCCATGC
p21-R	AAAGTCGAAGTTCCATCGCTC
MALAT1-F	CATTCGCTTAGTTGGTCTAC
MALAT1-R	TTCTACCGTTTTTAGCTTC
IGF2BP1-F	GCGGCCAGTTCTTGGTCAA
IGF2BP1-R	TTGGGCACCGAATGTTCAATC
MYC-F	TGCTCCATGAGGAGACACC
MYC-R	CTTTTCCACAGAAACAACATCG
LIN28B-F	CATCTCCATGATAAACCGAGAGG
LIN28B-R	GTTACCCGTATTGACTCAAGGC
STAT3-F	CAGCAGCTTGACACACGGTA
STAT3-R	AAACACCAAAGTGGCATGTGA
FTO-F	ACTTGGCTCCCTTATCTGACC
FTO-R	TGTGCAGTGTGAGAAAGGCTT
TIA1-F	CGAGATGCCCAAGACTCTATACG
TIA1-R	CCTTACCCATTATCTTCCGTCCA
dCasΦ-F	GAATCGCTTGGGATCGCAAC
dCasΦ-R	TTGGGATCAAGGCCGAAGTC
GAPDH-F	CGCTCTCTGCTCCTCCTGTTC

GAPDH-R	ATCCGTTGACTCCGACCTTCAC
